# Heart regeneration in adult *Xenopus tropicalis* after apical resection

**DOI:** 10.1186/s13578-017-0199-6

**Published:** 2017-12-13

**Authors:** Souqi Liao, Wenyan Dong, Luocheng Lv, Hongyan Guo, Jifeng Yang, Hui Zhao, Ruijin Huang, Ziqiang Yuan, Yilin Chen, Shanshan Feng, Xin Zheng, Junqi Huang, Weihuan Huang, Xufeng Qi, Dongqing Cai

**Affiliations:** 1 Key Laboratory of Regenerative Medicine, Ministry of Education, Jinan University, Guangzhou, 510632 People’s Republic of China; 2 Joint Laboratory for Regenerative Medicine, Chinese University of Hong Kong-Jinan University, Guangzhou, 510632 China; 3 International Base of Collaboration for Science and Technology (JNU), Ministry of Science and Technology, Guangzhou, 510632 Guangdong Province China; 4 Department of Developmental and Regenerative Biology, Jinan University, Guangzhou, 510632 China; 5 Stem Cell and Regeneration TRP, School of Biomedical Sciences, Chinese University of Hong Kong, Sha Tin, Hong Kong; 6 Institute of Anatomy, University of Bonn, Bonn, Germany; 7 Department of Molecular Genetics, Albert Einstein College of Medicine, Bronx, USA

**Keywords:** *Xenopus tropicalis*, Regeneration of cardiomyocytes, Heart injury and repair, Scar-free

## Abstract

**Background:**

Myocardium regeneration in adult mammals is very limited, but has enormous therapeutic potentials. However, we are far from complete understanding the cellular and molecular mechanisms by which heart tissue can regenerate. The full functional ability of amphibians to regenerate makes them powerful animal models for elucidating how damaged mature organs are naturally reconstituted in an adult organism. Like other amphibians, such as newts and axolotls, adult *Xenopus* displays high regenerative capacity such as retina. So far, whether the adult frog heart processes regenerative capacity after injury has not been well delineated.

**Results:**

We examined the regeneration of adult cardiac tissues of *Xenopus tropicalis* after resection of heart apex. We showed, for the first time, that the adult *X. tropicalis* heart can regenerate perfectly in a nearly scar-free manner approximately 30 days after injury via apical resection. We observed that the injured heart was sealed through coagulation immediately after resection, which was followed by transient fibrous tissue production. Finally, the amputated area was regenerated by cardiomyocytes. During the regeneration process, the cardiomyocytes in the border area of the myocardium adjacent to the wound exhibited high proliferation after injury, thus contribute the newly formed heart tissue.

**Conclusions:**

Establishing a cardiac regeneration model in adult *X. tropicalis* provides a powerful tool for recapitulating a perfect regeneration phenomenon and elucidating the underlying molecular mechanisms of cardiac regeneration in an adult heart, and findings from this model may be applicable in mammals.

**Electronic supplementary material:**

The online version of this article (10.1186/s13578-017-0199-6) contains supplementary material, which is available to authorized users.

## Background

Regeneration of the injured myocardium is a great challenge in clinical settings. It is well known that adult mammalian cardiac cells have very limited ability for cell proliferation. After cardiac injury, adult mammals, including humans, showed very limited ability for regeneration to replace the lost cardiomyocytes. The necrotic cardiac muscles are replaced with scar tissue, which will impair the contractility of the remaining myocardium, even cause heart failure and death if the injury is severe [1]. However recent studies indicated that mammalian cardiomyocytes are not postmitotic cells, indeed, mammalian cardiogenesis occurs during adult life, including in humans [2–7]. Thus, regeneration of the damaged myocardium is a tantalizing therapeutic goal. However, we are far from completely understanding the molecular mechanisms by which heart tissue can regenerate.

Lower vertebrates such as newts and zebrafish displayed extraordinary regeneration ability of cardiac tissue [8–10]. Anurans (frogs, toads) are another order of amphibians, within which it is known that frog tadpoles can regenerate their tails [11], and adult *Xenopus* have high regenerative capacity in retina [12, 13]. Cell proliferation of muscle fibers after heart injury was observed in adult frogs (*Rana temporaria*) [14]. However, whether the adult frog heart processes regenerative capacity after injury has not been well delineated.

The *Xenopus laevis* and *Xenopus tropicalis* are good models for studying embryonic development, and tissue regeneration. As they have unique advantage in the production of large eggs in abundant quantities throughout the year, the amenability of embryo to experiential manipulation, and the remarkable regenerative capacity of the tadpole and adult frogs [15–17]. The *X. laevis* has been one of the main animal models used for studying embryonic development, cell, electrophysiology, and biomedical studies [18–20]. However, *X. laevis* is an allotetraploid and has a long generation time, both of which hampers its application in genetic studies. In contrast to *X. laevis*, *X. tropicalis* has a smaller, true diploid genome. *X. tropicalis* exhibits 10 chromosome pairs containing 1.7 billion base pairs [21, 22], a shorter generation time [23, 24], and high synteny with the human genome [25]. We take advantage of the unique features of *X. tropicalis* to study the heart regeneration, and report for the first time that the adult myocardium of *X. tropicalis* can regenerate in a nearly scar-free manner following heart injury.

## Methods

### Experimental animals

*Xenopus tropicalis* frogs(Nigerian strain)were purchased from NASCO (USA), and maintained in a freshwater tank at 26 °C under a 12/12 light cycle. All the experimental protocol related with *X. tropicalis* was approved by the Jinan University Animal Care Committee.

### Apical resection of the X. tropicalis heart

**Fig. 1:**
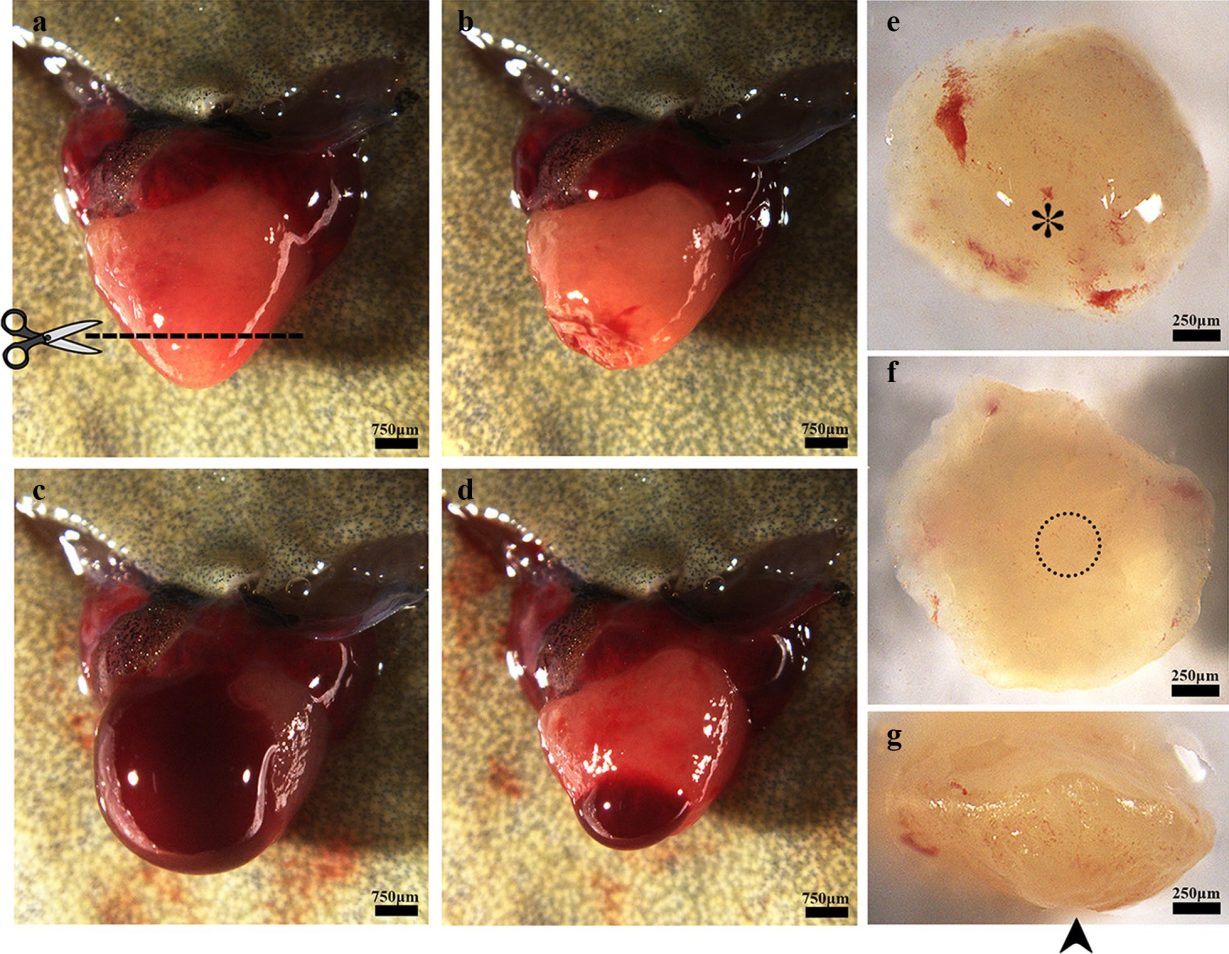
Apical resection of the *X. tropicalis* heart and rapid blood coagulation after resection. **a** The heart was exposed for apical resection. The dash line shows the resection plane, corresponding to approximately 10% of the ventricle. **b** The heart immediate after surgical amputation. **c** Bleeding after ventricle amputation. **d** Rapid blood coagulation in the amputated heart after approximately 5 s of pressure with sterile cotton. **e** Outer surface of the amputated apex. The asterisk indicates the end of the apex on the outer surface. **f** Inner surface of the amputated apex. The circle with the dotted line indicates the bottom of the ventricle. **g** Lateral view of the amputated apex. The arrow indicates the bottom of the apex on the outer surface

*Xenopus tropicalis* (female; 12 months old) were treated with Tricaine methanesulfonate bath (MS-222; 1 mg/mL; TCI, Shanghai, China) which was prepared with sterile double distill water at room temperature for 4 min and hibernated on ice for 60 s and then positioned ventral side up on an ice pad. The skin of the chest and upper abdomen was sterilized with iodine and 75% alcohol. A small incision was made near the heart using ophthalmic scissors. The pericardial sac was then opened, and the ventricle was exposed. Approximately 10% (approximately 1 mm in length) of the ventricle tissue from the cardiac apex was resected with Vannas scissors (Fig. 1a, b, e–g). The opened cavity was sutured with 4-0 suture after amputation. The *X. tropicalis* were subsequently transferred to and maintained in fresh water at 26 °C. To observe the survival rate and repeatability of the apical resection protocol, 65 *X. tropicalis* were observed. The 59 were survival after surgery of apical amputation, and six were died after surgery. The survival rate of the apical resection protocol is around 91%. Among fifty-nine survival *X. tropicalis*, thirty were included in present study. The animals were euthanized at 0 (approximately 30 min), 1, 2, 4, 8, 16, 30, and 60 days after apical resection (daar). The injured hearts were then isolated and rinsed with PBS (pH 7.4), followed by paraffin sectioning and staining. In this study, six frogs were observed in 30- and 60-day group, while three frogs were observed in 0, 1, 2, 4, 8, 16-day group.

### Hematoxylin–eosin staining

The injured hearts collected at different time points were fixed overnight in 4% paraformaldehyde, then dehydrated, cleared, and embedded in paraffin wax. Sections with a thickness of 5 μm were prepared for staining. The sections were deparaffinized in xylene (3 × 5 min) and rehydrated with successive 3-min washes in 100, 100, 100, 90, 80, and 70% ethanol with one final tap water wash. The sections were then stained with hematoxylin medium for 15 min, rinsed with tap water for 1 min, rinsed with 1% hydrochloric acid in 80% ethanol for 5 s, rinsed with a 1% ammonia solution for 5 s, and rinsed with tap water for 1 min. The sections were then stained with eosin medium for 3 min and rinsed with tap water for 1 min. After dehydration in an ethanol gradient and clearing with xylene, the slides were mounted with neutral balsam. The stained sections were observed and photographed under a microscope (Leica, Wetzlar GmbH DM4000B) with a CW4000 CCD camera controlled by Leica Application Suite software. For semi-quantitative analysis of red blood cells and inflammation in regenerated area, the images (20×) of regenerated area of all the time points were obtained. The density of red blood cells or inflammation respectively was set up to bottom level (+: a few scare red blood cells or inflammation cells in sham control myocardium which is matched to regenerated area of amputated heart,or in border area of wound at 0 daar myocardium), low (++: red blood cells or inflammation cells are infiltrated approximate 20–30% regenerated area), medium (+++: red blood cells or inflammation cells are infiltrated approximate 40–50 or 30–40% regenerated area) and high (++++: red blood cells or inflammation cells are infiltrated approximate 60–90 or > 50% regenerated area) as defined as Fig. 8a. The analysis for all the time points was conducted in double blinded method. Six animals were applied for 30 and 60 daar, while three animals were applied for other time points.

### Masson’s trichrome straining

Sections were prepared as described above. After deparaffinization and rehydration, the sections were stained with iron hematoxylin for 15 min, rinsed with tap water for 1 min, rinsed with 1% hydrochloric acid in 80% ethanol for 5 s, rinsed with 1% ammonia solution for 5 s, and rinsed with tap water for 1 min. The sections were then stained with Masson’s fuchsin-Ponceau mixture for 25 min and rinsed with tap water for 1 min. Next, the slides were treated for 10 min with phosphotungstic acid orange G and Masson’s light green solution for 4 min. After rinsing with tap water for 1 min, 1% glacial acetic acid was added for 1 min, and the slides were washed with tap water for 1 min. Dehydration in an ethanol gradient and clearing with xylene were conducted, and the slides were mounted with neutral balsam. The stained sections were finally observed and photographed under a microscope (Leica, Wetzlar GmbH DM4000B) with a CW4000 CCD camera controlled by Leica Application Suite software. For semi-quantitative analysis of fibrosis in regenerated area, the images (20×) of regenerated area of all the time points were obtained. The density of fibrous tissue production was set up to bottom level (+: a few scattered fibrous tissue production in border area of wound at 0 daar myocardium), low (++: fibrous tissue production is infiltrated approximate 20–30% regenerated area), medium (+++: fibrous tissue production is infiltrated approximate 40–50% regenerated area) and high (++++ : fibrous tissue production is infiltrated approximate > 50% regenerated area) as defined as Fig. 8a. The analysis for all the time points was conducted in double blinded method. Six animals were applied for 30 and 60 daar, while three animals were applied for other time points.

### Immunofluorescence

After deparaffinization and rehydration, heart tissue sections were subjected to heat-mediated antigen retrieval step using citrate buffer (pH 6.0) in a microwave oven. After antigen recovery, the sections were washed three times with PBS (pH 7.4) and blocked with 1% BSA for 45 min at room temperature. They were then incubated with a mixture of a phospho-histone H3 (Ser10) antibody (diluted 1:200 with 1% BSA; Cat No. 9701, CST, USA) and an anti-alpha skeletal muscle actin antibody (diluted 1:200 with 1% BSA; Cat No. ab49672, Abcam, UK) or an anti-cardiac troponin T (diluted 1:10 with 1% BSA; Cat No. CT3, DSHB, USA) at 4 °C overnight. After three washes with PBS, the sections were incubated with FITC-conjugated goat anti-mouse IgG (1:100; Cat No. SA00003-1, Proteintech, USA) and Cy3-conjugated goat anti-rabbit IgG (1:100; Cat No. SA00009-2, Proteintech, USA) for 45 min at room temperature. The sections were subsequently counterstained with DAPI and mounted with mounting medium. The stained sections were finally photographed using a fluorescence microscope (Leica, Wetzlar GmbH DM4000B) with a CW4000 CCD camera controlled by Leica CW4000 FISH software. For semi-quantitative analysis of mitotic cardiomyocyte, the images (20×x) of regenerated area of all the time points were obtained and the PH3^+^/α-SA^+^-positive cells were counted using double blinded method. The cell density in regenerated area ± standard deviation was used in semi-quantitative analysis. Three animals for each group were applied to analyse.

### Statistical analysis

The results are presented as the mean ± standard deviation (SD). All statistical analyses were conducted using the statistical software SPSS 19.0. One-way ANOVA with the less significant difference (LSD) test was used for intergroup comparisons. A value of P < 0.05 was considered statistically significant.

## Results

### The heart of adult *X. tropicalis* displays capacity of rapid blood coagulation after ventricle resection

The adult heart apical resection injury models used in this study were established by dissecting approximate 10% of the ventricle tissue from the cardiac apex (Fig. 1a, b, e–g). This resection damaged the ventricle wall. After resection, ventricle bleeding occurred immediately (Fig. 1c), and such bleeding could be stopped by applying pressure with sterile cotton for approximate 5 s. The heart beating maintained normal after the treatment for bleeding (Fig. 1d). The protocol established in the present study resulted in a survival rate of around than 91% (59 survival of the 65) after resection. In addition, all the *X. tropicalis* of 60 daar-group that underwent resection survived at least 60 days.

### The adult *X. tropicalis* heart has regeneration capacity after resection injury

**Fig. 2:**
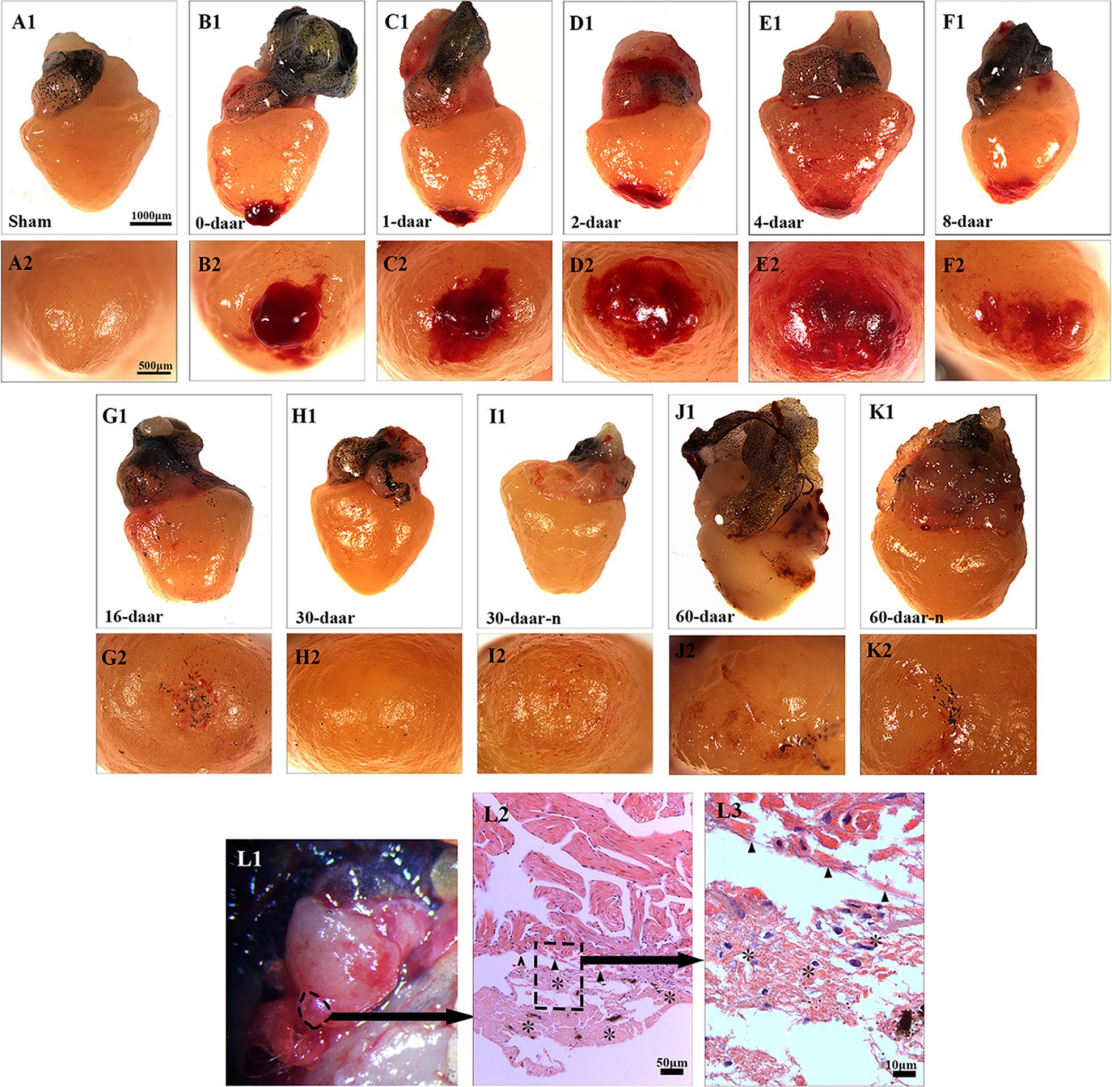
Regeneration of the injured *X. tropicalis* heart is demonstrated by morphology. **a1** A sham control heart. Bar = 1000 μm (the scale bar for **a1**–**k1**). **a2** The end of sham control apex on the outer surface. Bar = 500 μm (scale bar for **a2**–**k2**). The images illustrate the regeneration process of amputated heart at 0 daar (**b1**, **b2**, approximately 30 min after resection), 1 daar (**c1**, **c2**), 2 daar (**d1**, **d2)**, 4 daar (**e1**, **e2**), 8 daar (**f1**, **f2**), 16 daar (**g1**, **g2**), 30 daar (**h1**, **h2**; with a perfect regeneration morphology), 30 daar (**i1**, **i2**; with a nearly perfect regeneration morphology), 60 daar (**j1**, **j2**; with a perfect regeneration morphology) and 60 daar (**k1**, **k2**; with a nearly perfect regeneration morphology). **l1** Surface view of adhesion between the regenerated site and peripheral tissues in a nearly perfect regenerated heart at 60 daar (circle with the dotted line). **l2**, **l3** H&E staining showing adhesion between the regenerated site and peripheral tissues. High-power view of rectangle with a dotted line from **l2** (**l3**). Adhesion occurred between the regenerated site and peripheral tissues in the hearts that regenerated with a nearly heart-shaped morphology. However, an intact epicardial structure (small arrows in **l2**) was observed in the region between the regenerated myocardium and the adhesion tissue (asterisk). This observation suggests that the injured myocardium maintains the capacity to regenerate with a non-perfect morphology when the injured myocardium is affected by inflammation-associated adhesion. Six frogs were inspected for 30 and 60 daar, while three frogs were inspected for other time points respectively

The appearance morphology of regenerated *X. tropicalis* hearts after surgical injury was examine at 0, 1, 2, 4, 8, 16, 30, and 60 daar. At 16 daar, the exterior of the heart appeared normal, accompanied by the disappearance of inflammation and hyperemia; the injured cardiac apex of the ventricle was also nearly healed (Fig. 2a–g). At 30 daar, most of the experimentally injured hearts (approximately two-thirds) had regenerated with a perfect heart-shaped morphology (Fig. 2h), although some (approximately one-third) had regenerated with only a nearly heart-shaped morphology (Fig. 2i). To exclude the possibility that the nearly heart-shaped regeneration was due to an insufficient regeneration time, the injured hearts were further investigated at 60 daar. Similar to the results at 30 daar, we observed a part of injured heart (around two-third) is regenerated with perfect heart-shaped morphology in appearance (Fig. 2j), whereas approximately one-third regenerated with a nearly heart-shaped morphology (Fig. 2k). All of the injured hearts exhibiting perfect heart-shaped regeneration showed no adhesion between the regenerated site and peripheral tissues, whereas the injured hearts displaying nearly heart-shaped regeneration showed adhesion between the regenerated site and peripheral tissues (Fig. 2l).

**Fig. 3:**
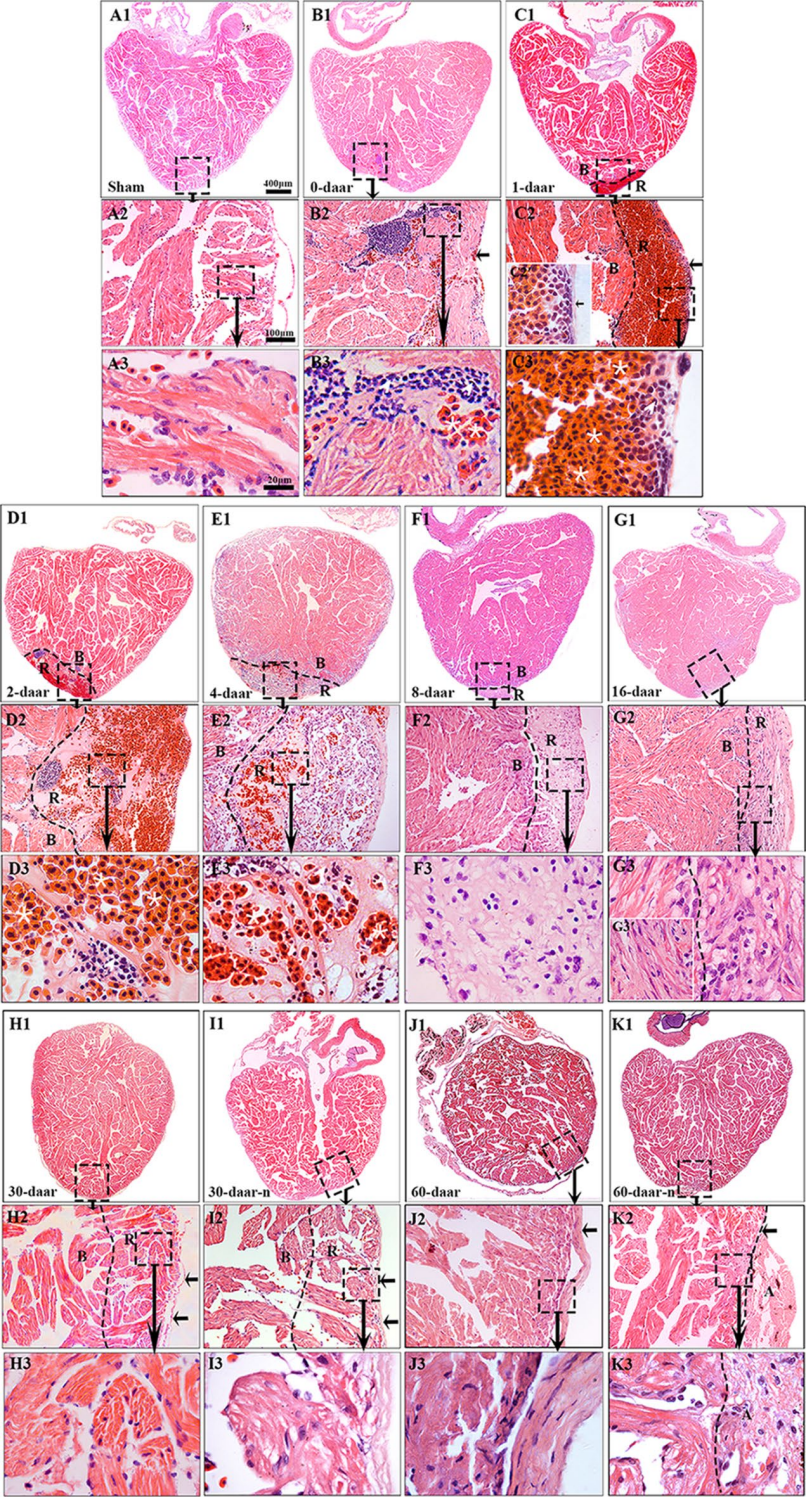
Adult *X. tropicalis* heart has capacity for regeneration after resection. **a1** Longitudinal section of an adult heart from the non-amputated sham control. Bar = 400 μm. **a2** High magnification of the region outlined by rectangle in **a1**. Bar = 100 μm. **a3** High magnification of the rectangle region in **a2**. Bar = 20 μm. **b1**–**b3** An amputated heart at 0 daar (approximately 30 min after amputation), showing activated inflammation and hyperemia at the border of the injury. **c1**–**c3** An amputated heart at 1 daar, showing a membrane-like structure (arrow) close to the outer surface of the scar tissue which exhibited with inflammatory cell infiltration, and red blood cells accumulation below the membrane-like structure. **c2′** High power of the membrane-like structure which is consisted of cellular or some extracellular matrix. **d1**–**d3** An amputated heart at 2 daar, showing more inflammatory cells in the regenerated area compared with 1 daar. **e1**–**e3** An amputated heart at 4 daar, showing an increase of inflammatory cells and a decrease of red blood cells in the regenerated area compared with 2 daar. **f1**–**f3** An amputated heart at 8 daar, showing the disappearance of most infiltrated red blood cells and a significant decrease of the inflammatory cell intensity in the regenerated area. **g1**–**g3** Most of the fibrous tissue production was replaced with newly regenerated cardiomyocytes and some of the newly regenerated cardiac myocytes are matured, as indicated by a more regular cardiac-specific cross-striated morphology at 16 daar (**g3′**). **h1**–**h3**, **i1**–**i3** An amputated heart regenerated with a perfect morphology and nearly perfect morphology at 30 daar. The amputated area was regenerated with mature cardiomyocytes. In addition, the regenerated myocardium has an intact epicardium (arrow). **j1**–j**3**, **k1**–**k3** An amputated heart regenerated with a perfect morphology and nearly perfect morphology at 60 daar. The amputated area was regenerated with mature cardiomyocytes. An intact epicardial structure (arrow) in the regenerated myocardium. *R* regenerated area, *B* border area. White star: showing red blood cells; White arrows: showing inflammation cells. Six frogs were inspected for 30 and 60 daar, while three frogs were inspected for other time points respectively

**Fig. 4:**
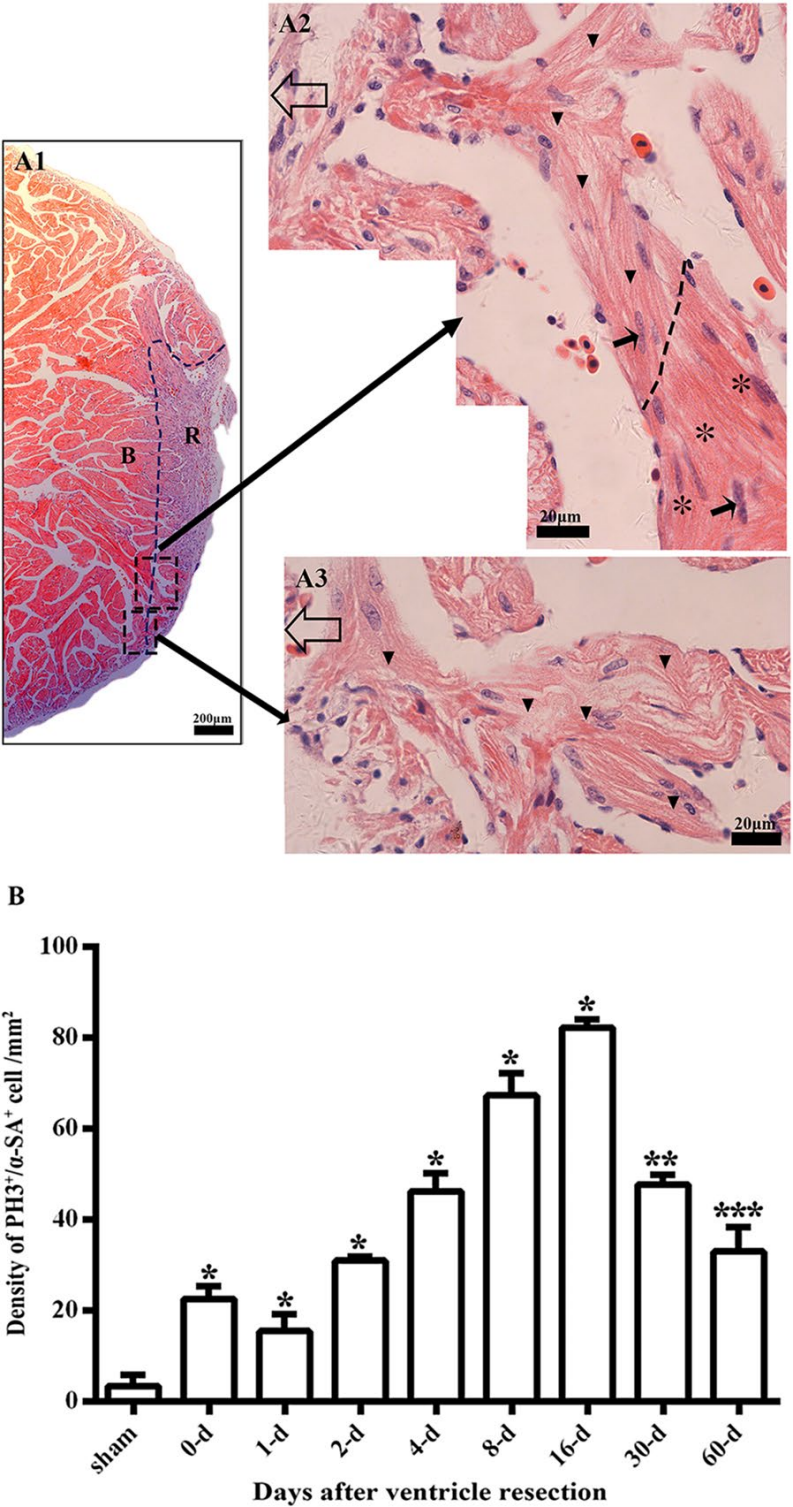
Cell proliferation and migrating morphology of cardiomyocytes during regeneration of the damaged myocardium. **a1** Longitudinal section of an amputated heart at 8 daar. *R* regenerated area, *B* border area. **a2** High magnification of large square with a dotted line from **a1**. Open arrow: epicardium site. Arrow: cardiomyocytes with dividing nuclei. Small triangle: regenerated cardiac myocytes, as indicated by light H&E staining, an irregular cross-striated structure, and migrating morphology in the regenerated site. Asterisk: endogenous mature cardiac myocytes, as indicated by intensive H&E staining and a regular cross-striated structure. **a3** High magnification of the small square indicated by a dotted line in **a1**. Open arrow: epicardium site. Small triangle: Regenerated cardiomyocytes. **b** Comparison of the density of α-SA^+^/PH3^+^ cardiac myocytes in different time points after apical amputation, which were from Fig. 5. *P < 0.01 vs. other groups. **P < 0.01 vs. other groups except 4-day. ***P < 0.01 vs. other groups except 2-day. The results of all time points are from three frogs, respectively

Histological analyses revealed some inflammation-associated cells and red blood cells on the proximal surface of the wound at 0 daar (Figs. 3b1–b3, 8) compared with non-amputated sham control heart (Fig. 3a1–a3). The red blood cells of *X. tropicalis* contain clear basophilic nuclei and eosinophilic cytoplasm, while the inflammatory cells can be recognized by their prominent basophilic nuclei and small cytoplasm. The wound site was filled with clot at 1 daar, and the outer surface of the clot tissue exhibited a membrane-like structure with some inflammatory cell infiltration and extracellular matrix. Many red blood cells accumulated to form a layer-like structure immediately below the membrane-like structure. In addition, many inflammatory cells had infiltrated the red blood cell layer (Figs. 3c1–c3, 8). At 2 daar, more significant inflammatory cell infiltration was observed in the clot, and most of clot was filled with extracellular matrix (Figs. 3d1–d3, 8). At 4 daar, in the regenerated area, the density of red blood cells was significantly decreased, and more inflammatory cell infiltration was observed when compared to those at 2 daar (Figs. 3e1–e3, 8). At 8 daar, most of the infiltrated red blood cells and inflammatory cells were disappeared in the regenerated area (Figs. 3f1–f3, 8). Some cardiomyocytes with dividing nuclei were found in the border area of the regeneration zone (Fig. 4a2). Numerous newly regenerated cardiomyocytes, characterized by light eosinophilic staining and an irregular cross-striated structure compared with mature cardiomyocytes, were found at the border of the regenerated area, some of which were characterized as showing a migrating like morphology toward the regenerated area (Figs. 4a1–a3, 6e). At 16 daar, most of the scar tissue was replaced with newly regenerated cardiomyocytes, and many of the newly regenerated cardiomyocytes had matured, as indicated by a more regular cardiac-specific cross-striated morphology (Fig. 3g1–g3). At 30 daar, we found that two-thirds of the amputated hearts (4 of 6) had regenerated with a perfect morphology, identical to normal control hearts, and the damaged myocardium had regenerated with a normal myocardial structure. However, one-third (2 of 6) of injured heart regenerated with a nearly perfect morphology that was nearly identical to the control hearts except the formation of adhesions between the newly formed myocardial tissue and peripheral tissue (data are only shown for the 60-daar group, not the 30-daar group). In the perfect regeneration group, all of the amputated areas were replaced by newly regenerated mature cardiomyocytes, as indicated by the regular striations. In addition, the epicardium had regenerated on the outer surface (Fig. 3h1–h3). However, in the nearly perfect regeneration group, the amputated area was replaced with newly cardiomyocytes, which were more mature compared with the regenerated cardiomyocytes at 16 daar. In addition, an intact epicardium was observed in the regenerated myocardium and included the adhesion area (Fig. 3i1–i3). The histological analyses of the 60-daar group further confirmed that most of the amputated hearts (approximately two-thirds [4 of 6]) regenerated with a perfect heart-shaped morphology (Fig. 3j1–j3), whereas approximately one-third (2 of 6) regenerated with a nearly heart-shaped morphology (Fig. 3k1–k3). All of the amputated hearts that regenerated with a nearly heart-shaped morphology contains adhesions between the regenerated site and peripheral tissues (Figs. 2l1–l3, 3k1–k3; data only shown for the 60-daar group, not the 30-daar group), whereas such adhesion was not observed in the amputated hearts that regenerated with a perfect heart-shaped morphology (Fig. 3j1–j3). However, in all of the nearly perfect regeneration groups (30–60 daar), epicardial structures were observed in the regenerated myocardium and in the area between the regenerated myocardium and adhesion tissue (Figs. 2l1–l3, 3k1–k3; data only shown for the 60-daar group, not the 30-daar group). This result suggests that the amputated myocardium maintained the capacity to regenerate in the nearly perfect regeneration group when the injured myocardium was affected by inflammation-associated adhesion. It demonstrated that the injured myocardium in adult *X. tropicalis* possesses regeneration potentials and is able to recover within approximately 30 days. However, the adhesion induced by inflammation might affect the regeneration of the amputated myocardium.

### The proliferation endogenous cardiomyocytes is an important mechanism of adult *X. tropicalis* heart regeneration

**Fig. 5:**
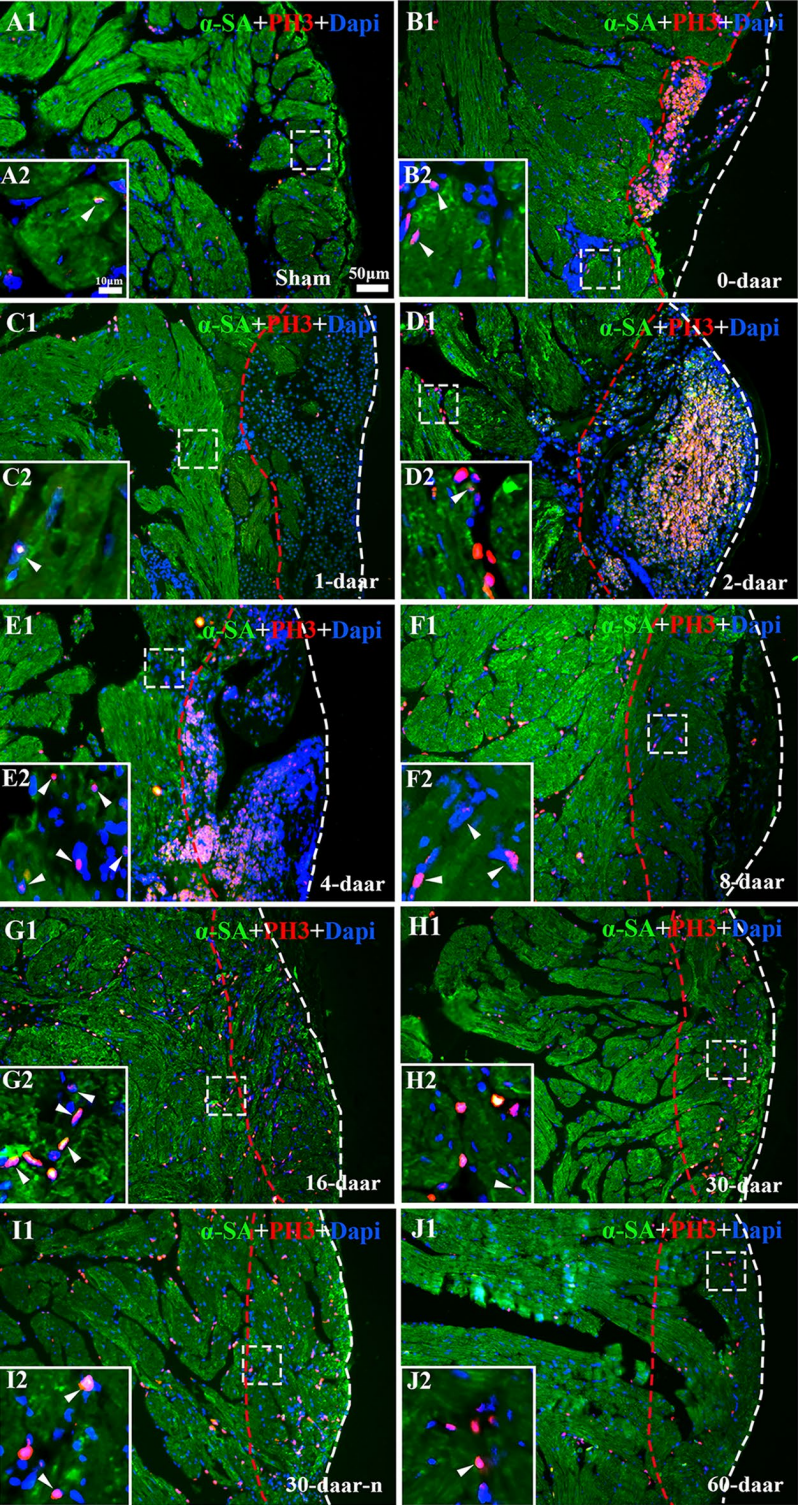
α-SA/PH3 positive cells exist in the border area of the amputated site and regenerated area. **a1** Longitudinal section of the apical area from a sham control. High magnification of the square region is shown (**a2**). Longitudinal sections of an amputated heart at 0 daar (**b1**, **b2**), 1 daar (**c1**, **c2)**, 2 daar (**d1**, **d2**), 4 daar (**e1**, **e2**), 8 daar (**f1**, **f2**), 16 daar (**g1**, **g2**), 30 daar (**h1**, **h2**; perfect regeneration), 30 daar (**i1**, **i2**; nearly perfect regeneration), and 60 daar (**j1**; perfect regeneration**)**. The amputated apex was regenerated by newly cardiomyocytes within approximately 30 days after amputation, as demonstrated by the presence of many α-SA^+^ cardiomyocytes in the regenerated zone (area between the red dotted line and white dotted line). In addition, the density of α-SA^+^/PH3^+^ cardiac myocytes in the border area of the regenerated zone at 2–8 daar and in the regenerated zone at 16–30 daar is significantly higher than those in the sham control and in 0–2 daar groups, suggesting that the proliferation of endogenous cardiomyocytes in the border area of the amputated site might be an important mechanism for regeneration of the damaged myocardium. *α-SA* alpha skeletal muscle actin, *PH3* phospho-histone H3, *DAPI* 4′,6-diamidino-2-phenylindole. White arrow: PH3^+^ nucleus. White dotted line, outer surface of the epicardium. Area between the red dotted line and white dotted line, regenerated area after amputation. 30-daar-n: amputated heart with nearly perfect regeneration at 30 daar. Bar in **a1**–**j1** = 50 μm. Bar in **a2**–**j2** = 10 μm. Six frogs were inspected for 30 and 60 daar, while three frogs were inspected for other time points respectively

**Fig. 6:**
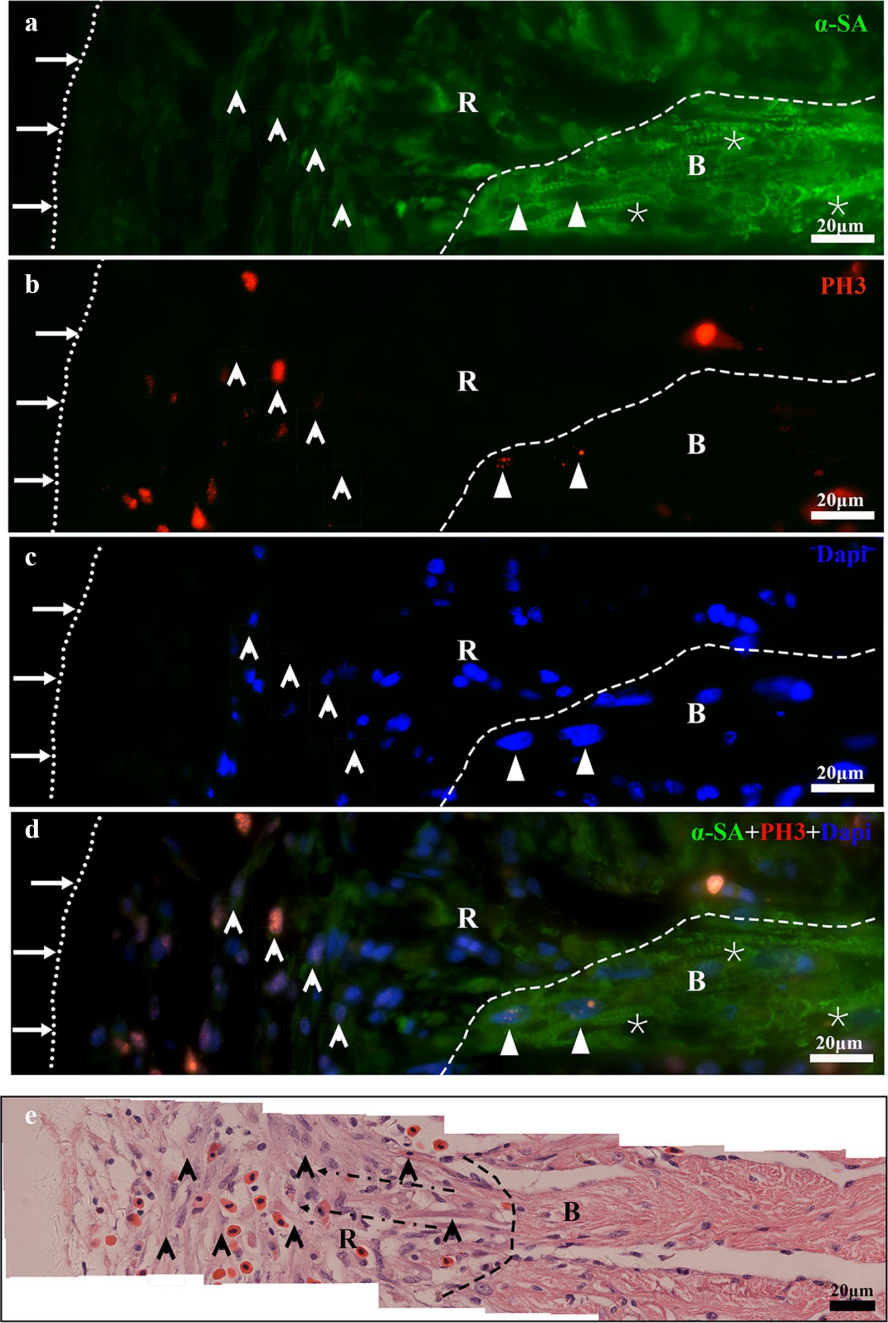
Immunofluorescent staining combined with H&E staining indicating the proliferation of cardiac myocytes during regeneration. **a**, **b** Immunofluorescent staining for α-SA^+^(**a**) and PH3^+^ (**b**) in a same section of the regenerated zone at 8 daar. Nuclei were counterstained with DAPI (**c**). The overlay image is shown in (**d**). **e** H&E staining of section from near dimension of A. Long arrow and small dotted line: Outer surface of the epicardium. Short arrow: Newly regenerated α-SA^+^/PH3^+^ cardiomyocytes (**a**–**d**) and newly regenerated cardiomyocytes in H&E staining (**e**). Small triangle: Mature α-SA^+^/PH3^+^ cardiac myocytes. Asterisk: mature cardiac myocytes with a regular cross-striated structure. Black arrow with dotted line: Migrating morphology of cardiomyocytes (**e**). Large white black dotted line: Border of the regenerated zone. *R* regenerated area, *B* border area. The results are from three animals

The cellular mechanism underlying the regeneration of adult *X. tropicalis* cardiac tissue was further investigated. Double-fluorescence staining of PH3 and alpha skeletal muscle actin (α-SA^+^), was performed to identify mitotic cardiomyocytes. PH3 labels mitotic cells and α-SA is a marker for cardiomyocytes. A basal level of PH3^+^/α-SA^+^-positive cells was observed in normal cardiomyocytes (Figs. 4b, 5a1, a2). However, the density of PH3^+^/α-SA^+^-positive cells was significantly increased in regenerated area and near the wound area between 4 and 16 daar. The peak of PH3^+^/α-SA^+^-positive cells was observed between 8 and 16 daar (Figs 4b, 5f1, f2, g1, g2; *P* < 0.01). At 4 and 8 daar, some PH3^+^/α-SA^+^-positive cardiomyocytes with a disorganized cytoskeletal morphology were found within the regenerated area, and many PH3^+^/α-SA^+^-positive spindle cells were found in the regenerated area located within the area of epicardial tissue (Figs. 5e1, e2, f1, f2, 6a–d). At 30–60 daar, the density of PH3^+^/α-SA^+^-positive cells in the regenerated myocardium had significantly decreased (Figs. 4b, 5h1, h2, j1, j2). Between 30 and 60 daar, the amputated area was regenerated by newly cardiomyocytes with mature cardiac phenotypes as indicated by regular cardiac-specific cross-striated morphology and cardiac troponin T staining (Fig. 3h1–h3, k1–k3 and Additional file 1: Figure S1H1, H2, J1, J2). Furthermore, the histological results shown in Figs. 4a and 6e also suggest that the proliferation of endogenous cardiomyocytes play an important role in the regeneration of the amputated apex. All of these observations suggest an important role of the proliferation of endogenous cardiomyocytes during regeneration of the injured adult *X. tropicalis* myocardium and the amputated myocardium was regenerated with mature cardiomyocytes in around 30 days.

### The regeneration of the injured adult *X. tropicalis* heart is nearly scar-free

**Fig. 7:**
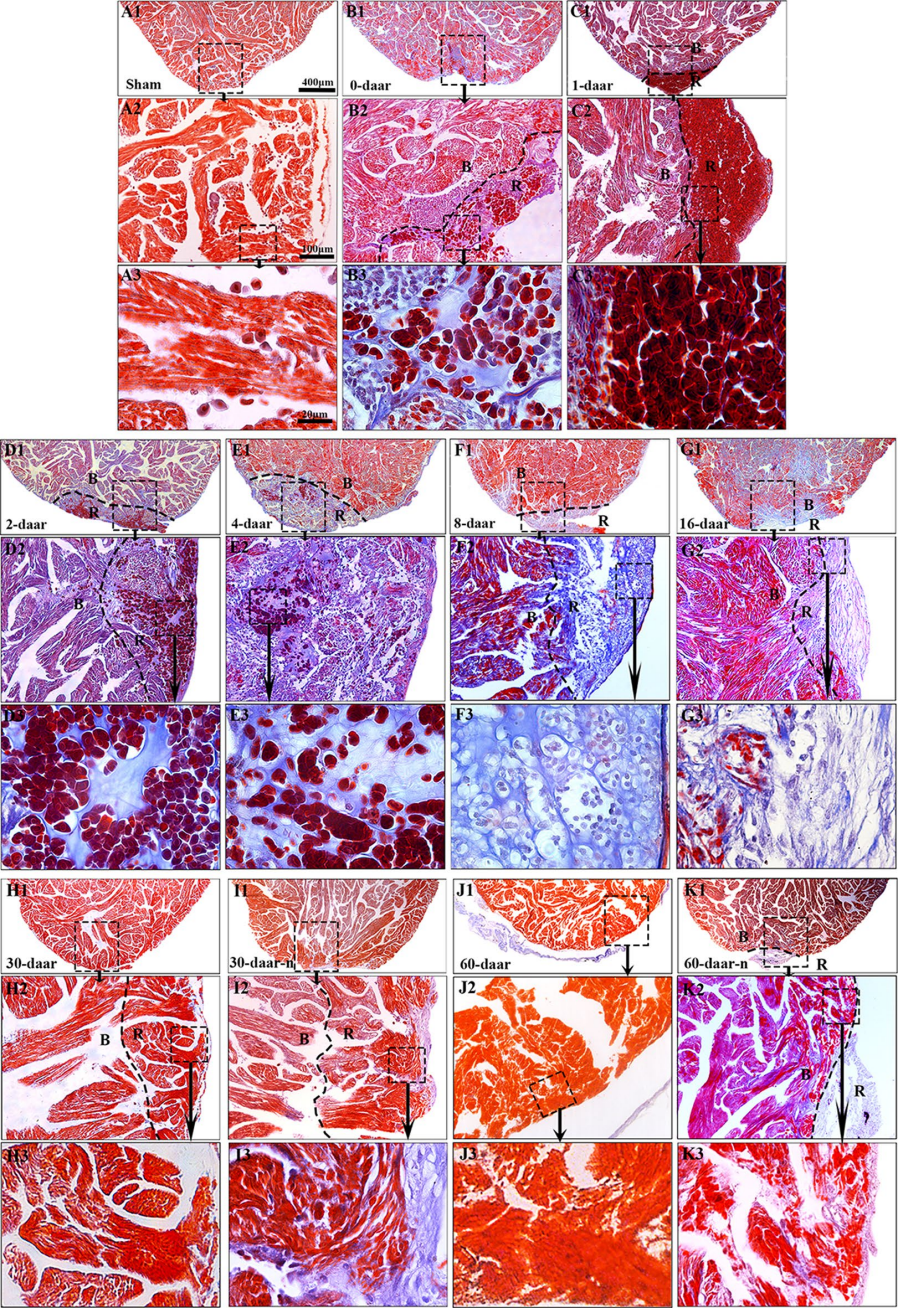
The regeneration of the injured adult *X. tropicalis* heart is nearly scar-free manner. **a1** Longitudinal section of an adult heart from a sham control. Bar = 400 μm (scale bar for **a1**–**k1**). **a2** High magnification of the rectangle with a dotted line from **a1**. Bar = 100 μm (scale bar for **a2**–**k2**). **a3** High magnification of rectangle with the dotted line from **a2**. Bar = 20 μm (scale bar for **a3**–**k3**). Longitudinal sections from amputated heart at 0 daar (**b1**–**b3**), 1 daar (**c1**–**c3**), 2 daar (**d1**–**d3**), 4 daar (**e1**–**e3**), 8 daar (**F1**–**f3**), 16 daar (**g1**–**g3)**, 30 daar (**h1**–**h3**; an amputated heart regenerated with a perfect morphology), 30 daar (**i1**–**i3**; an amputated heart regenerated with a nearly perfect morphology), 60 daar (**j1**–**j3**; an amputated heart regenerated with a perfect morphology**)** and 60 daar (**k1**–**k3**; an amputated heart regenerated with a nearly perfect morphology). Fibrosis-like structures were stained in blue. In the hearts that were regenerated with a nearly perfect morphology, fibrosis-like structures was only observed in adhesion tissue, but not in the regenerated myocardium between 30 and 60 daar. *B* border area. *R* regenerated area. Six frogs were inspected for 30 and 60 daar, while three frogs were inspected for other time points respectively

Scar formation is a barrier to the perfect regeneration of the mammalian myocardium. Transient fibrous tissue production was observed during myocardium regeneration in *X. tropicalis*. The results obtained through Masson’s trichrome staining revealed that a collagen deposition and fibrous tissue production were present in regenerated area at 0–16 daar (Figs. 7a1–a3, g1–g3, 8). The highest intensity of fibrous tissue production was found at 4 daar, after which fibrous tissue production decreased progressively from 8 to 30 daar (Figs. 7e1–e3, i1–i3, 8). At 30 daar, the fibrotic structure was rarely observed in the fully regenerated myocardium (Figs. 7h1–h3, i1–i3, 8). This observation was further confirmed at 60 daar, when fibrotic tissue was rarely found in the regenerated myocardium in the perfect heart-shaped or nearly heart-shaped regenerated hearts. In addition, in nearly heart-shaped regenerated hearts, fibrotic tissue was only found within the peripheral adhesion tissue and not in the regenerated myocardium (Fig. 7j1–j3, k1–k3). Taken together, injured adult *X. tropicalis* heart tissue shows high degree of regeneration capacity, and can regenerate nearly scar-free myocardium.

**Fig. 8:**
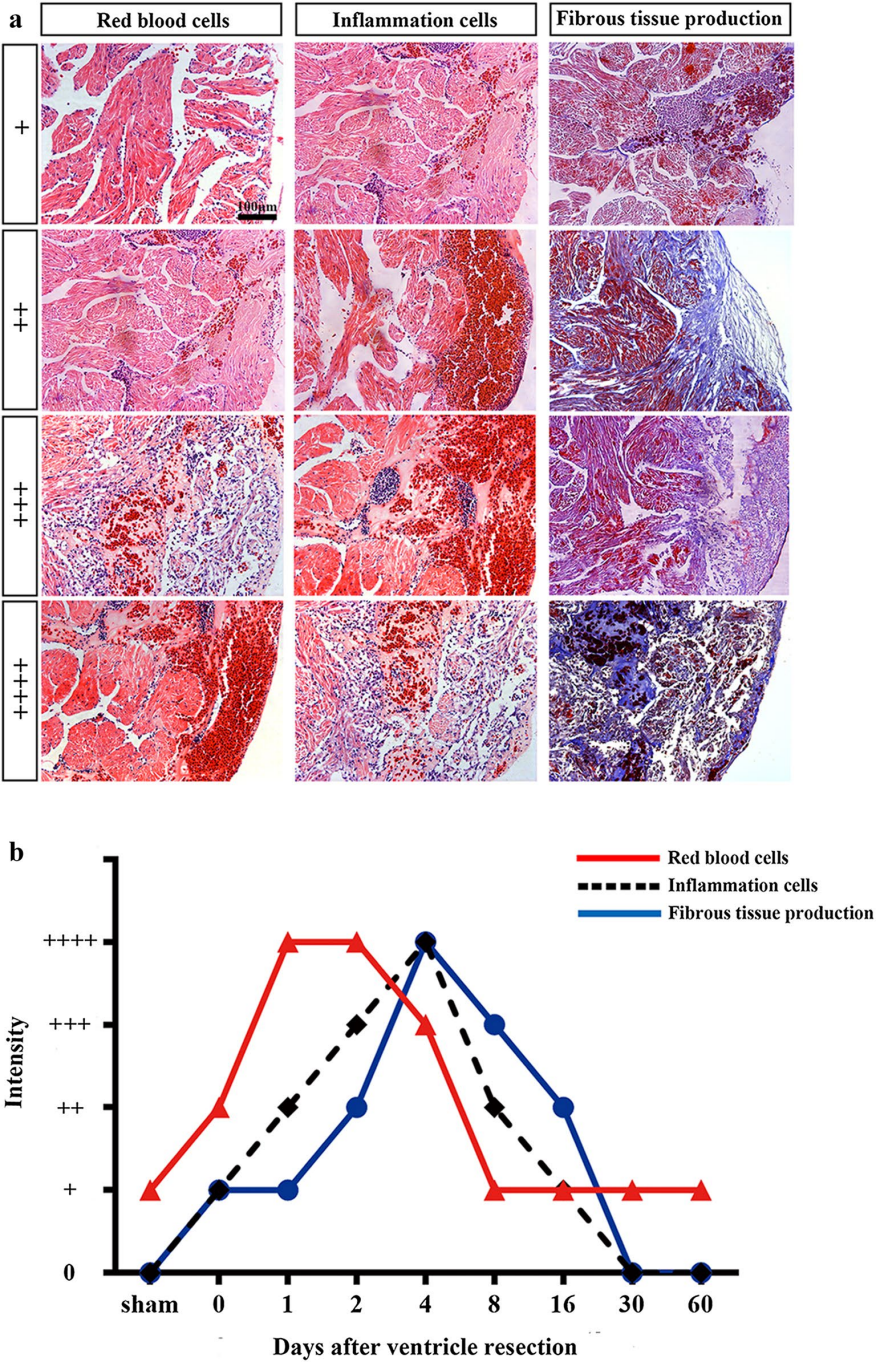
Time course of infiltration of red blood cell and inflammation, and fibrosis during the regeneration of amputated myocardium. **a** Images represent various degrees of infiltration of red blood cell and inflammation cells, and fibrous tissue production during heart regeneration. **b** Comparison of time courses for infiltration of red blood cell and inflammation cells, and fibrous tissue production

## Discussion

In this study, we demonstrate that the injured adult *X. tropicalis* heart can be regenerated in a nearly scar-free manner within approximately 30 days after the resection of 10% apical of the cardiac apex. Our results clearly showed that within 30 days after amputation, the cardiac apex of the ventricle was regenerated by mature cardiomyocytes, as revealed by α-SA staining (a marker of cardiomyocytes), cardiac troponin T (a marker of mature cardiomyocytes) and the striation of cardiomyocyte. Furthermore, the regenerated myocardium was covered with epicardium. Importantly, we found that when no inflammation-associated adhesion occurred, regeneration of the *X. tropicalis* heart could be achieved in a nearly scar-free manner, with normal morphology and histological structure. In addition, the survival of injured *X. tropicalis* up to 60 days after resection suggest that the regenerated myocardium exerts normal physiological functions.

The rapid blood coagulation after ventricle amputation seems important for *X. tropicalis* heart regeneration. The surgical protocol used here damaged the ventricle wall and caused ventricle bleeding, which can result in death in mammals. However, we found that ventricle bleeding could stop by applying pressure to the wound for approximate 5 s after amputation, and the amputated heart maintained normal beating without bleeding when the pressure was released. Importantly, the injured *X. tropicalis* were able to survive after resection. In addition, we observed rapid coagulation of red blood cells in the injured area. Many red blood cells accumulated and form into a layer-like structure, which might function as a “dam-like stuffing” at the periphery of the wound site at 0, 1, and 2 daar. Accordingly, we speculate that red blood cell accumulation at the periphery of the wound might participate in clotting to facilitate blood coagulation after ventricle resection. It is possible that the red blood cells are cleared when the ventricle bleeding stop and the regeneration of cardiac tissue is triggered. In line with this hypothesis, we found that at 8 daar and thereafter, most red blood cells were disappeared from the regenerated area, and many regenerated cardiomyocytes were found in the regenerated area. It is well established that damage of cardiomyocytes results in a high risk of cardiac bleeding and even rupture in the beating mammalian heart. Therefore, mammals utilize rapid scar healing to reduce risk of cardiac rupture after myocardium damage. The findings of the present study might suggest that the loss of rapid coagulation in mammals might be an important reason for the scar healing process observed in the mammalian heart.

Inflammation is a critical initiation step for wound healing and regeneration [26, 27]. We found that inflammatory cells infiltrated the wound within approximately 30 min after resection, and the highest numbers of these cells were observed approximately 4 days after apex amputation before progressively returning to a normal physiological level. The pattern of the inflammation response observed in *X. tropicalis* is quite similar to the normal wound-healing pattern observed in other species, such as fish and mammals [28]. This finding suggests that the inflammatory response to wound healing and regeneration of the heart in *X. tropicalis* is conserved. In addition, the disappearance of red blood cell infiltration was concurrent with the obvious decline of inflammatory cells in the regenerated area at 8 daar. Yet the underlying biological significance remains elusive.

We also investigated whether the proliferation of cardiac myocytes is a key mechanism for the regeneration of damaged cardiac myocytes. We found that between 2 and 16 daar, numerous PH3-positive myocytes were present in the regenerated area. At 8 daar, many newly regenerated cardiac myocytes-characterized by light eosinophilic staining, an absence of cross-striated structure, and α-SA staining were found in the regenerated area, exhibiting a migrating morphology. At 30 daar, the newly regenerated cardiac myocytes become mature, as indicated by regular cardiac-specific cross-striated morphology and cardiac troponin T staining. Immunofluorescence staining for both PH3 and α-SA showed that the density of PH3^+^/α-SA^+^-positive cells significantly increased. These cells were mainly localized at the border of the regenerated area from 2 to 16 daar. These results suggested that the proliferation of endogenous cardiac myocytes are important mechanisms for the regeneration of the *X. tropicalis* myocardium after apical resection. We were not able to investigate whether the adult cardiac stem cell plays a role in the regeneration of the *X. tropicalis* myocardium because of lack of reliable markers and antibodies.

In this study, we found a moderate fibrous tissue production in regenerated area in the injured heart between 2 and 16 daar. However, the fibrotic structure decreased progressively. At 30 daar, when the injured heart was fully regenerated with an intact myocardium, the fibrotic structure was rarely observed in the regenerated area. All of these results suggested that the adult *X. tropicalis* heart maintains the scar-free regeneration capacity. Understanding the molecular mechanism that regulates scar free regeneration will help to establish novel strategies to repair damaged mammalian myocardium. Our research also suggests that adhesion caused by inflammation can affect morphological reconstruction and prevent a perfect regeneration pattern. This finding further confirms that an appropriate level of inflammation and the prevention of pathological adhesion are evolutionarily conserved mechanisms facilitating healing and regeneration.

*Xenopus tropicalis* is a good model for heart regeneration. The injury protocol for the adult *X. tropicalis* heart established in the present study achieved a survival rate was greater than 90%. In addition, all the *X. tropicalis* of 60 daar-group that underwent resection survived at least 60 days. The injured hearts were recovered by scar-free regeneration. Thus the established model will be a powerful tool for studying the mechanisms of cardiomyocyte regeneration and developing novel strategies to regenerate injured mammalian cardiac tissue.

## Conclusions

Present study established the heart apex resection and regeneration model and showed, for the first time, that adult *X. tropicalis* heart can be regenerated in a nearly scar-free manner after resection injury. Rapid coagulation involving red blood cells is crucial to cease ventricle bleeding, maintain the survival and initiate the regeneration of the injured myocardium. The cardiomyocyte proliferation appears important for the myocardium regeneration. The established model set up an excellent platform for studying heart regeneration.

## Additional file

**Additional file 1: Figure S1.** Cardiac troponin T^+^ and Cardiac troponin T^+^/PH3^+^ cells exist in the regenerated area between 8 to 60 daar. A1: Longitudinal section of the apical area from a sham control. High magnification of the square region is shown (A2). Longitudinal sections of an amputated heart at 0 daar (B1–2), 1 daar (C1–2), 2 daar (D1–2), 4 daar (E1–2), 8 daar (F1–2), 16 daar (G1–2), 30 daar (H1–2; perfect regeneration**)**, 30 daar (I1–2; nearly perfect regeneration), and 60 daar (J1; perfect regeneration). Cardiac troponin T^+^ and Cardiac troponin T^+^/PH3^+^ cells existed in the regenerated area between 8 to 60 daar. The amputated apex was regenerated by mature cardiomyocytes as indicated cardiac troponin T positive within approximately 30 days after amputation. cTnT: Cardiac troponin T; PH3: Phospho-histone H3; DAPI: 4′,6-Diamidino-2-phenylindole. White arrow: PH3^+^ nucleus. White dotted line, outer surface of the epicardium. Area between the red dotted line and white dotted line, regenerated area after amputation. 30-daar-n: Amputated heart with nearly perfect regeneration at 30 daar. Bar in A1 to J1 = 30 μm. Bar in A2 to J2 = 10 μm.

## Abbreviations

*X. tropicalis*: *Xenopus tropicalis*
*X. laevis*: *Xenopus laevis*
daar: days after apical resection
PH3: phospho-histone H3
α-SA: alpha skeletal muscle actin
cTnT: cardiac troponin T
DAPI: 4,6-diamidino-2-phenylindole
